# More Than Fish—Framing Aquatic Animals within Sustainable Food Systems

**DOI:** 10.3390/foods11101413

**Published:** 2022-05-13

**Authors:** Alexandra Pounds, Alexander M. Kaminski, Mausam Budhathoki, Oddrun Gudbrandsen, Björn Kok, Stephanie Horn, Wesley Malcorps, Abdullah-Al Mamun, Amy McGoohan, Richard Newton, Reed Ozretich, David C. Little

**Affiliations:** 1 Institute of Aquaculture, University of Stirling, Stirling FK9 4LA, UK; a.m.kaminski@stir.ac.uk (A.M.K.); mausam.budhathoki@stir.ac.uk (M.B.); bjorn@blonkconsultants.nl (B.K.); s.j.horn@stir.ac.uk (S.H.); wesley.malcorps@stir.ac.uk (W.M.); a.mcgoohan@sms.ed.ac.uk (A.M.); richard.newton@stir.ac.uk (R.N.); r.w.ozretich@stir.ac.uk (R.O.); d.c.little@stir.ac.uk (D.C.L.); 2 Department of Clinical Medicine, University of Bergen, 5020 Bergen, Norway; oddrun.gudbrandsen@uib.no; 3 Department of Fisheries and Marine Science, Noakhali Science and Technology University, Noakhali 3814, Bangladesh; mamun@nstu.edu.bd; 4 The Royal (Dick) School of Veterinary Studies, University of Edinburgh, Midlothian EH25 9RG, UK

**Keywords:** aquaculture, fisheries, human nutrition, micronutrients, planetary boundaries, sustainability

## Abstract

Aquatic animals are diverse in terms of species, but also in terms of production systems, the people involved, and the benefits achieved. In this concept piece, we draw on literature to outline how the diversity of aquatic animals, their production, and their consumption all influence their impact within the food system. Built on evidence from an array of reductionist and non-reductionist literature, we suggest that food systems researchers and policymakers adapt current methods and theoretical frameworks to appropriately contextualise aquatic animals in broader food systems. We do this through combining current understandings of food systems theory, value chain, livelihoods, nutritional outcomes, and planetary boundaries thinking. We make several claims around understanding the role of aquatic animals in terms of nutritional output and environmental impacts. We suggest a need to consider: (1) the diversity of species and production methods; (2) variable definitions of an “edible yield”; (3) circular economy principles and the impacts of co-products, and effects beyond nutrient provision; (4) role of aquatic animals in the overall diet; (5) contextual effects of preservation, preparation, cooking, and consumer choices; (6) globalised nature of aquatic animal trade across the value chain; and (7) that aquatic animals are produced from a continuum, rather than a dichotomy, of aquaculture or fisheries. We conclude by proposing a new framework that involves cohesive interdisciplinary discussions around aquatic animal foods and their role in the broader food system.

## 1. Introduction

Aquatic animals are globally important to many populations [1]. Traditionally sourced from wild stocks, aquatic animals are increasingly supplied through aquaculture, a sector that is rapidly growing to meet rising global demand. Today, aquatic animals from both fisheries and aquaculture are critical to nutritional security for many vulnerable groups [2,3,4,5,6]. In 2015, fish accounted for 17% of animal protein globally, providing a substantial part of daily animal protein for over 3 billion people [6]. Regions with some of the highest per capita intake of aquatic animals, such as small island states, are also areas where other animal source foods are relatively expensive and scarce [6]. Nutritionally, aquatic animals have been valued as a source of not only protein, but a range of micronutrients, including some, such as DHA and EPA n-3 PUFA fatty acids, that are only found in aquatic animals and algae [6]. Aquatic animals support nutritional security directly through consumption, and indirectly through both income generation and livelihoods that support diverse diets. Associated value chain systems linked to both fisheries and aquaculture also support non-fishing, non-farming livelihoods [7,8,9,10] through spill-over effects within the broader community [11].

**Figure 1:**
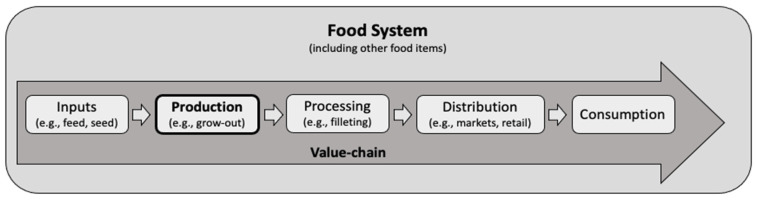
Aquatic animal production is a node within a value chain and broader food system.

Aquatic animal production is only one part of the larger aquatic animal value chain, embedded within the broader food system (Figure 1). Aquatic animal value chain systems contribute to a complex food system that is critical to attaining the Sustainable Development Goals (SDGs) [12,13]. While aquatic foods contribute to broader development across the SDGs, aquatic animals as food fall directly under two main SDGs: SDG 14 (Life Below Water) stresses the need to reduce environmental impacts on marine environments, whereas nutritional outcomes are encompassed by SDG 2 (Zero Hunger). Yet, in dividing the role of aquatic animals under these two perspectives, we risk mis-framing aquatic ecosystem health versus food security as a trade-off [14], missing nuanced relationships between nutrition, livelihoods, and the environment. For example, does reducing fishing intensity negatively impact on the nutritionally vulnerable and if so, what are effective and affordable mitigating actions? Can aquaculture fill the demand gap with affordable and accessible products, or should we be looking beyond aquatic foods to ensure nutritional security for all?

Secure food systems, both terrestrial and aquatic, rely on natural ecosystem services that need to be well-managed, both now and in the future, so that food systems can be truly ‘sustainable’. ‘Sustainable diets’ are defined as “human consumption habits that are protective and respectful of biodiversity and ecosystems; culturally acceptable; accessible; economically fair and affordable; and nutritionally adequate, safe and healthy, while optimising natural and human resources” [15]. Improving human nutritional outcomes with reduced environment impacts has become a major theme in the broader food security literature [16,17,18,19,20,21], often aligned around the principle of ‘planetary boundaries’ [22].

Aquatic animals, both wild-caught and farmed, tend to have lower environmental impacts than terrestrial animal source foods [23,24]. The claim is that aquatic animals are important for achieving both food security as well as more sustainable food systems [12,25]. Despite their important contributions, aquatic animals have been side-lined or ignored in higher-level discussions and policies around ‘food security’ [26]. Numerous calls have been made for closer integration of aquatic products in human nutrition research [14,27,28,29,30]. Aquatic animals have been characterised using a variety of terms, including ‘aquatic foods’, ‘fish’, ‘seafood’ or most recently, ‘blue foods’, reflecting different perspectives. For example, ‘blue foods’ and ‘seafood’ align with an ‘ocean’ narrative, but aquatic animals come from all water bodies and aquaculture has developed most significantly in freshwater rather than the sea [31,32,33,34]. In reality, the Blue Economy/Ocean narrative is incomplete in several respects [14,32]. Most obviously, even fish production occurring in the ocean is intimately connected with both terrestrial and freshwater inland environments through flows of feed, nutrients, and water [31]. In this paper, we use the term ‘aquatic animals’ to encompass the array of animal species that are supplied from both freshwater and marine environments. Narratives around aquatic foods increasingly emphasise the importance of farmed plants, but these remain marginal on a global scale and only quantified for marine and brackish water systems [31]. Seawater macroalgae and freshwater vegetables can be important on a local scale in terms of livelihoods and nutrition respectively, but here we focus on aquatic animals.

The contextualization of aquatic animals’ role in broader food security and sustainability has only recently come under greater scrutiny following the release of the Blue Food Assessment [10,12,13,31,35]. Previous reductionist attempts to evaluate the role of aquatic animals in terms of broader food security and sustainability did not consider the diversity of aquatic animals, the variety of their production systems, and the nuances of how aquatic animals affect diets, livelihoods, and the environment [36]. Nutritional value to humans, livelihood implications, and environmental impacts are frequently assessed in isolation and rarely considered together [29,37,38]. While reductionist science is critical and valuable, this lack of multidisciplinary cohesion makes identification of trade-offs and potential solutions difficult to achieve at a policy level. Bridging the aquatic disciplinary bubble remains a challenge.

In this paper, we argue that gaps exist in discussions on aquatic animals for human consumption, which have yet to be clearly conceptualised in terms of food systems. These gaps are evident from the lack of disciplinary cohesion within the literature and arise from holistic food systems discussions drawing on select reductionist scientific literature [39]. While reductionist science is critical for understanding the effects of select variables, it leads to these gaps when poorly contextualised. These gaps have led to misconceptions, myths, and false narratives as to aquatic animals’ function in reaching the above-mentioned goals. Policy discussions concerning aquatic animals tend to be individually centred around the various disciplinary perspectives mirroring reductionist literature, in particular biological ‘productionist’ approaches that view food systems within narrow productivity parameters [40] rather than broader food systems outcomes [41]. Researchers and policymakers have yet to define a robust framework that incorporates all relevant dimensions specific to the nuances of aquatic animal foods [42].

In this paper, we aim to identify the gaps, question claims, and propose grounds for new and improved relationships between constructs and theories to better understand the role of, and implications for, aquatic animals in sustainable food systems. Based on the following issues, we explain the misconceptions and myths in the current narratives around aquatic animal systems. We indicate which points are in reference to the entire value chain, and which are specific to the point of production. Then, we suggest how each aspect could be perceived more realistically, using current understandings of food systems theory, value chains, livelihoods, nutritional outcomes, and planetary boundaries thinking. Finally, we propose a new framework that addresses these limitations for understanding and improving aquatic animal food systems. New approaches must acknowledge the diversity of production systems, the variety of species, and societal roles and outcomes. They must also be interdisciplinary, considering the contextualised nutritional value, environmental and social impacts of all production outputs.

## 2. The Issues

### 2.1. The Diversity of Aquaculture

Research on the impacts of broader food systems has, until recently, either disregarded the major differences between aquatic animals and other animal-source foods e.g., [21], or has aggregated them into a single ‘fish’ category alongside the major terrestrial species e.g., [17,19]. Some aquaculture- and livelihoods-specific studies quantify ‘fish’ consumption in terms of total fish consumed by weight or value, but do not consider the variation in nutritional content occurring between species e.g., [5,11,43,44,45,46]. In general, the contribution of aquatic animals to nutritional outcomes in dependent populations is vastly understudied [27].

Disaggregating aquatic animals into more diverse categories is important to understand their nutritional and environmental impact [47]. It is well-understood in separate reductionist literature that the environmental impacts and nutritional value of the product for consumers vary by species and production system [6,14]. Permutations of species and production systems technology can have different impacts and outcomes [48]. As a result, environmental impacts and nutritional outcomes can also vary substantially, making ‘global’ aggregated data unreliable at local levels. For example, consider carp species, of which the majority are Grass carp (*Ctenopharyngodon idella*), Silver carp (*Hypophthalmichthys molitrix*), Common carp (*Cyprinus carpio*), Bighead carp (*Hypophthalmichthys nobilis*), and Catla (*Catla catla*). These species made up over 38% of global production by volume in 2018 and remain important in diets throughout much of Asia [1]. In contrast, the introduction of Silver carp and Bighead carp as exotics threatens some American freshwater ecosystems [49]. The relationship between the species and the context is critical for understanding impact.

The diversity within aquaculture is enormous: global reviews report over 1700 species of aquatic animals harvested and over 620 of aquatic animal species cultured, including finfish, crustaceans, molluscs, and amphibians [1], although the majority of production consists of approximately only 10 species [31]. Aquaculture is an immature sector compared to other food sectors and in general, the production of most species has not consolidated around a single culture system or species [50]. The very diversity of systems and species may be a key element of the food systems’ resilience, particularly as many remain in the domain of small-scale actors [10,50].

Production systems are diverse: they include cages, ponds, or raceways for culturing fish or crustaceans; submerged ropes for culturing bivalves; and set nets, trawled nets, hook and line, or traps for harvesting wild fish. Some systems are intensive, requiring nutritionally balanced formulated feeds and other inputs to maintain water quality [51]. Feed, other inputs, and a range of other factors, impact on a variety of global and/or local environmental parameters, including carbon emissions, water use, land footprint and local pollution from sludge (including faeces, feed leftovers, some residual medications, etc.) [12,23]. The production and use of aquafeed ingredients is responsible for a majority of the overall environmental impact of fed-aquaculture production through its value chains: feed ingredients that supply the fish with essential macronutrients (e.g., fats and proteins) may originate both from marine (i.e., fish meals and oils) or terrestrial sources (e.g., soy, rapeseed) and have their own specific associated environmental impacts [23,48,52,53]. Farmed fish were initially fed on diets containing large quantities of small pelagic fish, particularly anchovies; however, due to economic incentives and, increasingly, environmental and biodiversity concerns, terrestrial plant-based ingredients have been gradually substituted for marine ingredients. The shift from marine ingredients towards terrestrial crop-based ingredients has inevitably added pressure to agricultural production systems [54,55,56], which are already under pressure to meet demand for food, feed, biofuel, and biobased materials [57]. While reduction in marine ingredients from poorly managed fisheries will likely result in positive biodiversity impacts, such as those modelled for Chinese fisheries [34], well- (or acceptably) managed fisheries, such as for most anchoveta and other key species, produce ingredients for aquafeeds that have lower carbon footprints compared to the major plant replacements [53]. For example, uncertified Brazilian soy protein concentrate has a carbon footprint an order of magnitude higher than anchoveta when considering land use change along with the associated concerns of deforestation and habitat loss [53]. Intensive crop production often requires higher use of land, pesticides, and fertilisers, which can lead to terrestrial ecotoxicity and eutrophication in freshwater and marine ecosystems [53,58]. Aquatic animal production systems vary in degree and range of environmental impacts.

Other culture systems are extensive, relying on natural foods growing on background levels of nutrients, or semi-intensive systems that boost yields through use of fertilisers to encourage natural food in situ sometimes with direct supplementary feeding. Despite no feed inputs, extensive systems can have high land requirements requiring more clearance of natural vegetation and loss of carbon-rich topsoil, such as when shrimp culture ponds replace mangrove forest [59]. Polycultures are the norm in Asian extensive and semi-intensive systems, where the co-production of multiple crops complicates evaluating the individual impacts of each product. In these types of systems, input variables are influenced by uncontrollable externalities, where the ‘boundary’ of the system may be difficult to define. For example, runoff from agriculture can impact the water quality of coastal mussel production [60].

The feed and water quality of the culture environment affects the nutritional profile of both fed and non-fed species. Consider Atlantic salmon (*Salmo salar*), valued for its high concentration of long-chain n-3 polyunsaturated fatty acids (n-3 PUFA). Substituting the aforementioned long-chain-n-3-PUFA-rich marine ingredients with plant ingredients in aquafeeds has negative consequences on the nutritional outcome of the final product [61,62,63,64]. The n-3 PUFA content of farmed salmon decreased by 50% between 2006 and 2015 as a result of substituting terrestrial ingredients for marine ingredients in salmon aquafeed [62]. The n-3 PUFA levels in tilapia are also dependent on feed formulations [65] with important health implications for vulnerable groups of consumers [66]. Feed formulation studies generally evaluate growth rates and efficiency ratios and rarely consider specific nutritional outcomes for consumers [27].

As an example for unfed species, consider that both mussels [67] and tilapia [65,66] cultured in different systems demonstrate a wide range of nutritional profiles depending on a range of factors—but primarily the type and quality of their feed. Blue mussels (*Mytilus edulis* L.), usually grown on ropes, have different nutritional composition [49] depending on the season and sex: in winter, phospholipids—fatty acids that reduce the risk of coronary heart disease and cancer [68]—account for 12% of lipids in female mussels and 34% of lipids in male mussels, compared to in summer, where they account for 37% of lipids in females and 28% in males [69].

Tilapia are produced in a range of production systems and intensities, as diverse as feeding on natural food only in earthen ponds and rice fields through to being fed complete formulated diets in cages in lakes or rivers. Size at harvest is also highly variable, affecting feed conversion ratio (FCR—a major indicator of environmental impact) and nutritional value [70]. Production of tilapia and carp fingerlings (small, immature fish) in rice fields is not only highly efficient in terms of FCR, but also contributes towards the farmers’ nutritional security [71]. Free-breeding tilapias have become important for vulnerable populations wherever they have become established, both in fisheries [72] or managed in culture systems [73]. As discussed further below, the complex nature of how tilapia are cultured and consumed challenges comparative assessments between species based on published nutritional compositions of fillets, in which tilapia perform relatively poorly e.g., [13].

### 2.2. What Is the Edible Yield?

A fillet is often used as a standard measure of ‘edible yield’, and yet, an ‘edible portion’ extends beyond the fillet yield in many contexts; definitions of ‘edible portion’ are both context- and culture-specific, and subject to change over time. Global modernisation of food cultures around supermarkets and consumption of processed aquatic animals, usually as fillets, compared to ‘traditional’ food cultures, is itself an oversimplification [74]. Even in Europe and North America, where fillets are popular, certain viscera products, such as swim bladder, milt, and roe, are popular foods among some consumer groups.

In the literature, definitions and understandings are frequently dominated by a narrow interpretation of an ‘edible portion’ e.g., [12,29]. This is problematic because the nutrient contribution of aquatic animal products varies based on what part(s) of the animal is consumed, as different parts of the animal have different nutritional profiles [62,65]. The consumed portion, and hence nutritional value, often varies greatly between individuals: food is portioned and consumed differently between communities and even within the household [75]. Roos [76], recognising this limitation, modified her nutritional intake studies in Bangladesh after observation of normal fish cleaning and preparation practises. Roos [76] demonstrated that identifying and defining ‘edible yields’ of aquatic animals is critical in assessing their nutritional value and the potential food security outcomes from their consumption. Unfortunately, we lack nutrient composition data for many species as well as for the various parts of each species; nutrient levels are typically assessed based on fish samples consisting of fillets or the whole fish, disconnected from local consumption norms. Faced with a lack of data, fish consumption studies submit to using the harvested weight of the fish or weight of the fillet. Some studies round portion sizes to the nearest 100 g of parts of the fish that are frequently consumed (e.g., fillets, heads, tail, etc.). The lack of data also limits the impact of nutrient composition databases, such as the FishBase nutrition-modelling database [77], which aggregates and models data including both whole fish and fillet portions from multiple sources.

A complete understanding of the nutritional contribution of aquatic animal production will require a broader ‘systems thinking’ perspective, especially considering the global trade of processed seafood co-products. Commonly found where fish are highly processed, ‘by-products’ are secondary co-products generated incidentally alongside the primary co-product (and the economic driver for production), typically the fillet or tail (for crustaceans). An excellent example of these can be found during the processing of the salmon farmed in Scotland, Norway, and Chile; when the fish is separated into fillets and into other co-products, including heads, frames, and belly flaps. Salmon by-products often have low demand in higher value markets, and so are therefore sold in international markets; salmon heads are exported to Vietnam, frames to Eastern Europe, and belly flaps are highly regarded in Japan [78]. For the salmon industry in Norway, most opportunity for value increase could be by adding value to underutilised by-product resources; however, the profitability of the fillet for other fish species is still the main driver of production.

Disregarded, ‘non-edible’ by-products are often still retained within a food system responsive to economic incentives: a common use of unsorted fish trimmings (by-products of filleting) has been as an ingredient in animal feeds, including for pets, [79] and in marine ingredient (fishmeal and fish oil) production. Although there has been considerable effort to utilise by-products from seafood processing more effectively, by-product use varies considerably by region. Many Asian value chains commonly retain these resources, effectively resulting in little waste. In contrast, European legislation—in response to bovine spongiform encephalopathy and other food scares—has made utilisation more difficult [41,80]. A mixture of incentives and tax burdens (particularly landfill tax) in Europe are pushing companies towards better utilisation of these by-products [41,79,81,82], which has opened up new industries around marine ingredients, human consumption, health products, and industrial uses, including for leather and cosmetics [79,82,83,84]. In other areas where by-product value is low and there are few opportunities and few penalties for inappropriate disposal, challenges remain. Better targeting of markets for such by-products can improve the proportion used as direct human food and the overall value of production [79,85]. The important nutritional contribution of these increasingly utilised by-products might be missed if the analysis is only framed around the fillet.

### 2.3. Broader Benefits from Production Systems

Nutrition and public health literature considering aquatic animals tend to focus on a particular species and/or nutrients. While valuable in its focus, this reductionist approach requires broader contextualisation: the multifaceted co-production of species and products (aquatic and terrestrial) produced from these systems affects food security and public health outcomes. Chinese small-scale inland ponds are a traditional example of aquatic animals co-produced in food systems [86,87] that continue to support surrounding vegetable production on the pond dykes, irrigated by the pond [88]. Such systems are now a key feature of contemporary food production elsewhere in Asia, where pond aquaculture is well established and has become critical for household and local community’s food security, for example, in Bangladesh [89] and Vietnam [90]. Co-production of foods must be considered to understand their cumulative nutritional effects, including the flow of nutrients through the broader food system, and environmental effects. For example, ensuring aquaculture discharge water becomes a horticultural input rather than a pollutant requires informed design [91] and, typically, local-level collaboration between producers [92]. Nutritional and environmental impacts ideally need a food-system-wide assessment rather than drawing boundaries around the immediate value chain or production segment.

Policymakers and development agencies often overlook the wide range of ecosystem services that aquatic animal production systems provide. Filter-feeding bivalves and certain fish (e.g., Chinese silver carp, *Hypophthalmichthys molitrix*) can improve water quality through their filtration and consumption of micro-particulates, which could contribute to regional carbon-trading markets [93]. Failed aquaculture initiatives can produce ecological refuges with both positive and negative impacts on human health and the surrounding environment [94]. For example, a fish pond can become a refuge for nutritious, self-recruiting species [95,96], but can also be a breeding ground for mosquitos that can increase local rates of malaria and other mosquito-borne illnesses [97]. The potential benefits to public health through predation of snail vectors of schistosomiasis by molluscivorous fish [98] or mosquitos by larvivorous species [99] are examples of how production can provide health services to local communities beyond nutrition or livelihoods [100]. Many households also use their local ponds for domestic use (cleaning and bathing), with significant welfare and hygiene implications [43].

These benefits need to be mapped to fully understand and capture the efficiency and benefits of the food system, as ignoring co-products and ecosystem services could lead to overestimates of negative environmental impacts of production [101] and underestimates of the nutrition and health benefits.

### 2.4. Emergent Methodologies for Measuring Environmental and Nutritional Outcomes

Assessing the environmental impact of nutritional targets is a long-held aspiration but raises a number of challenges. Although nutritional assessments could be made in parallel to environmental assessments, they should be integrated using different methodologies into an LCA framework that allows trade-offs to be assessed on an equivalent basis. Early LCAs often tried to compare certain foods based on a single nutritional characteristic e.g., [102], such as a quantity of protein as the “Functional Unit” (FU) (i.e., the reference unit of the assessment) e.g., [103]. Dietary quality scores have usually been made against a reference unit such as mass of food, protein, or energy content e.g., [103]; however, this and other articles [104] have demonstrated that food (and seafood especially) provides much more benefit to the consumer than any single macro- or micro-nutrient, and in varying amounts. A key consideration of any LCA is what the reference unit is appropriate for, in the measuring of impacts. When comparing between products, LCA methodology requires that the function of products is assessed, which becomes problematic when comparing food with varying quantities of different nutrition and, therefore, function. Although some authors have attempted a combined analysis of the environmental and nutritional effects of aquatic animals, combined assessments are not routinely adopted for any food product. Incorporating the complexity of nutrition into LCA is inevitably challenging [105].

Recent efforts have focused on developing a methodology around nutrition as an impact category, encompassing nutritional profiling of foods into a dietary quality scoring system so that they can be characterised in a similar manner to environmental impacts and directly integrated into an LCA framework [106]. Several different methods have received interest for further development and integration [106]. Typically, approaches adopt a system of reference to dietary guidelines or recommended intake of nutrients which may be considered positive (e.g., vitamins) or negative (e.g., saturated fat) using a series of indicators. However, approaches to calculating guidelines for consumption differ; for example, percentage of energy intake for macronutrients compared to Recommended Daily Allowances (RDA) for micronutrients. RDA guidelines were used as the basis for a study environmental and nutritional impact of food waste in the UK [107] and for a global study of environmental and nutritional trade-offs [108]. Quantification of qualifying or disqualifying substances in relation to RDAs or Maximum Recommended Intake respectively further complicates matters, where overconsumption of key nutrients does not further enhance health benefits or even becomes a health issue [106]. While several nutrition papers discuss the concept of “capping” such nutrients at a certain level, there are significant challenges of how such concepts can be integrated into an LCA framework. Other considerations include the availability of nutrients and interaction between different nutrients contained within a food product or meal, and therefore whether they have the same nutritional value. Scoring nutritional intake, positively or negatively, then becomes subjective compared to regional or national intake guidelines for different nutrients and how they are consumed. Potentially, the mechanisms by which different characterisation factors are applied for environmental impacts, such as water scarcity factors in the AWARE method [109] and in the ReCiPe eutrophication impact category [110] might be used; for example, to how RDAs could be adjusted for different nutrients in different countries and contexts.

Over twenty different nutritional scoring systems for foods were identified by [106], but only a few could reasonably be integrated consistently with LCA methodology. Environmental impacts within LCA are separated into several categories. Key considerations include whether nutritional content should be presented as single or several characterised impact categories and whether positive and negative nutritional characteristics should be presented separately. Impact categories/indicators could then be compiled into an index, although local, regional, and demographic contexts will apply to scoring and weighting, reflecting the nutritional status of different populations. However, in any case, a substantial effort is required to characterise the wide array of nutrients into a comprehensive database. The substantial challenge of characterising nutritional factors within an LCA framework has received much consideration through various approaches. One of the leading approaches is to apply the ‘Disability Adjusted Life Years’ (DALY) methodology [18,102]. DALY is a well-established methodology within the Recipe LCA methodology for assessing toxicological effects. The principle of relating DALYs to nutrition is underpinned by the availability of data on dietary quality indices linked to health outcomes. Typically, risk ratios for different diseases caused by dietary factors are used to calculate DALYs e.g., [111] as part of the Global Burden of Disease (GBD) reports [18,102]. Since the early approaches of applying risk factors to DALYs, the methodology has been continually improved. More factors have been included to develop the DALY Nutritional Index, which incorporates both qualifying and disqualifying nutrients into a single scoring system for Combined Nutrition and Environmental (CONE) LCA [18].

Most environmental assessments, including LCA, only reference ‘live fish at farm gate’ and rarely consider processed products. Even when processed products are evaluated, often only the primary product is considered, without assessing the utilisation of the whole animal. This has implications for the array of micronutrients within co-products, which are rarely considered. Many studies only consider the nutritional content of the fillet, failing to consider differences in the ‘edible portion’ [13], as discussed above. Even the new Blue Foods Assessment has published environmental and nutrition impacts of aquatic foods in separate papers on environmental performance [112] and nutrition [13]: the nexus of environmental impact and nutritional value of aquatic foods in relation to broader human diets at a nutrient and aquatic-species-specific level remains undeveloped at both global and local contexts.

A further limitation concerns the availability and quality of secondary data underpinning both LCAs and related to the nutritional content of aquatic animals as consumed e.g., [12,13,27,31,112]. Life cycle inventory data for most fish-dependent populations in the developing world are generally lacking. Most LCAs have been conducted for European and American markets [113], and the nutritional value of products as consumed also fails to consider the diversity of different production and consumption systems. Combining nutritional and environmental outcomes becomes most useful in well-defined contexts where sufficient data are available, such as examples from Sweden [29] and Bangladesh [38]. This type of interdisciplinary approach requires appropriate methodology, interpretable indicators, and high-quality data. Food composition databases (FCDB) are essential for this type of research; however, data on fish are still lacking from the major FCDBs [114]. There is an urgent need to collect and collate food composition data to be able to assess the nutritional outcomes of food systems [114,115]. Understanding the diversity of ways in which aquatic animals contribute to nutrition and diets is discussed in detail in a later section.

Some studies conclude that the ‘most nutritious species’ generally also have the lowest carbon footprints, such as bivalves [13,20,103], but this research fails to consider the importance of production systems variability within species. Even species grown in standardised systems can have variable nutritional content, as explained in the first section. Attempts at assessing environmental impacts of any aquatic food in the context of human nutritional needs must also consider affordability and nutritional relevance, particularly for vulnerable populations [16]. The specific roles of each aquatic food depend on its context in the broader diet and interactions with other dietary components. Food items that may seem ‘nutrient-poor’ when considered in isolation, may actually impact positively on health outcomes due to their accessibility, quantities consumed, and nutritional complementarity with other foods consumed. For example, tilapia has been assessed as having a low nutritional value per 100 g of edible portion compared to other species e.g., [116], but such simplistic assessment based on nutrient density alone ignores accessibility and affordability. A lower price point may support more frequent consumption of larger portions and higher overall nutrient intakes than ‘more nutritious’ options.

An analysis by Hallstrom et al. [29] did combine food systems dimensions to appropriate and species-level inclusion of aquatic animals in a defined context (Sweden) but is not generalisable beyond such boundaries. Similarly, modelling by Shepon et al. [38] is also an exemplary comparison of nutritional outputs of various aquaculture systems in comparison with nutritional composition of fish from fisheries, and their methods should be applied to contexts beyond Bangladesh. These methodologies are useful models, but fall into the same traps discussed throughout this paper in disregarding affordability, system co-products, consumption behaviours, and preferences. This is possibly due to the use of secondary data: these two studies were mostly desk-based and aimed to generalise. Now that the methodology is becoming more advanced, future studies can, and should, focus on collecting high-quality primary data specific to each context.

### 2.5. Contextual Differences: Delivering Nutritional Benefits from Aquatic Foods

The importance of aquaculture, particularly in poorer countries, has traditionally been framed around providing animal protein to diets deficient in this key macronutrient e.g., [117]. In recent years, attention has been drawn to its importance in stemming a ‘hidden hunger’ for micronutrient deficiencies, as discussed above. Micronutrient deficiencies are a result of nutritionally insufficient diets. A nutritionally sufficient diet is one that contains adequate recommended daily allowances of both macro- and micro-nutrients, where micronutrients are supplied through a wide variety of foods. Assessing how specific aquatic foods contribute to diets that are nutritionally sufficient requires understanding the broader nutritional needs of the community. For example, the n-3 PUFA level may be meaningful in diets of populations where obesity and cardiovascular diseases are a public health issue, whereas levels of calcium and Vitamin B12 may be relatively more important in contexts where dairy and terrestrial meat are less accessible or less consumed (e.g., low-income demographics). Yet, messages around single nutrients, be they n-3 PUFAs, individual minerals, trace elements, or vitamins, can oversimplify a nutritional message of how aquatic foods generally supply a cocktail of micronutrients.

We must use such reductionist research on the contribution of individual nutrients in combination with contextual understandings of food culture. These elements include food pairings and the compound effect of nutrients from the broader diet, cooking and preservation methods, and food safety, which we will now discuss in turn.

1. Food pairings and compound effects: Aquatic animals can supply an array of bioavailable, commonly deficient micronutrients for humans [2,4], a property that is of even greater value when considered as part of the total diet. For example, the co-consumption of fish can increase the bioavailability of certain nutrients in vegetables [75,118]. In addition, traditional recipes may call for certain ingredients to be paired with a fish, that have direct nutritional impacts on the consumer as well as affect the bioavailability of nutrients in the fish (e.g., cod and bacon is a popular food pairing in Norway, and Peruvian ceviche includes lime juice with a white fish such as sea bass or tilapia). As mentioned above, methods by Weidema and Stylianou [18] may be one way of modelling the cumulative effects of these types of food pairings.
2. Cooking and preparation methods: The effect of aquatic animal consumption on diet-related health outcomes may vary with how the food is prepared, portioned, and cooked [2]. Available cooking methods alter people’s ability to consume and digest aquatic animals and the nutrition available with possible trade-offs [119,120]. For example, drying small fish softens the bones such that young children can eat the whole fish and benefit from the calcium-rich content [121]; however, drying the fish may also alter its amino acid profile [122]. Baked and canned sardines also contain bones that are edible. Drying, fermenting, or canning may alter the nutritional value of fish. Beyond altering the nutritional value of the product, different production, preparation, or preservation methods can affect health aspects negatively. Preservation chemicals, such as salt and pigments may negatively affect human health, as can contaminants and adulterants post-harvest; yet, preservation methods can prevent spoilage, supporting nutritional needs during lean periods [123]. Furthermore, such processing can increase the affordability of fish while still providing some health benefits, such as the n-3 PUFA content of tinned mackerel, typically much cheaper than fresh alternatives [124]. Regulations that support safe and clean post-harvest practices are critical, particularly given the importance of processed fish in contexts like sub-Saharan Africa, where cold chains are short and cultural tastes lean towards preserved products [125]. The cultural and livelihood implications of processing—an activity often dominated by minority or marginalised groups—as well as the role of processed fish in broader food security are understudied; most literature on processed fish concerns its aesthetic or food safety properties [126].
3. Food safety: Nutrition outcomes are influenced by food handling and safety: sanitation practices (i.e., WaSH) and other food safety initiatives have positive effects on disease mitigation [127] and enhanced absorption of nutrients from foods [128]. Aquatic animals and their environments are often central to the transmission of diseases, such as those caused by food-borne trematodes [129], and have been associated with accumulation of contaminants in the food chain [130,131]. Different aquatic species [132] and production systems [133,134] can influence safety and quality aspects of the consumed product; public mis-information that farmed aquatic animals have more contaminants than those from the wild has threatened international trade and food security [135]. Moving from a ‘food safety’ perspective towards a ‘One Health’ lens [136] may be a good basis for integrating environment, human, and animal health. This system framework may also encourage a move away from consumers’ unbalanced focus on negative outcomes associated with aquatic systems such as heavy-metal toxicity, antimicrobial resistance, and plastic contaminants [137].

For aquatic animals to have any effect, consumers must choose to include them in their diet. Drivers for consumer choice and, in turn, nutritional outcomes, are complex [42,138,139], but fundamentally are underpinned by affordability, access, and cultural preferences [13,139,140,141]. Consumers may also have demands around quality and organoleptic properties, which may be altered by different production systems (chemical, sensory, flavor, safety; [142]). Different demographics’ food choices may have different drivers [138]. While a few studies on western consumers show a preference for fish with ecolabels [143], consumer choices are mainly driven by price rather than sustainability credentials [144], where consumers’ preferences (aspirational) may differ from their actual practices [145]. Ensuring sustainable practices will likely rely on legislation and certification of products [146,147,148]. Yet, the importance of sustainability also varies by region [149]. Chinese retailers tend to show a relatively higher interest in messaging around “safety” and “quality” (i.e., health benefits and natural characteristics of seafood); in contrast, retailers in Europe and the USA tend to show a higher interest in “sustainability” messaging, driven by demand for ecolabels and sustainable production practices [149]. The role for governance in supporting sustainable production practices is critical in light of these conflicting drivers for consumer choice.

Dietary transitions that accompany lifestyle changes can also affect nutritional outcomes. A shift in affluence or move from rural to urban lifestyles that makes convenience a higher priority, for example, can lead to greater consumption of ready-to-eat or snack formats, for example, as in China [88]. The cultural norms around food consumption, whilst diverse, are also dynamic in relation to emerging opportunities [150]. An example of these types of opportunities is the recent yet rapidly growing demand for imported farmed salmon among the Asian middle class. A parallel rise in diversity in aquaculture production is growing to meet this type of demand [151]. Another example is rapid variations in the target markets for Vietnamese pangasius tilting from the US to the EU, and more recently, to China [135,152].

The relevance of aquatic animals in the diet is also impacted by social norms [153,154]. There are many populations and individuals globally who eat no aquatic foods. Some social categories are entirely absent from fish value chains. For example, high-caste Brahmins in India religiously abstain from fish production and consumption, in contrast to unscheduled castes and tribes [155]. Another example is people practising Judaism, who refrain from the consumption of shellfish or fish without scales. Even within demographics with high rates of fish consumption, not everyone eats aquatic animals. Vulnerable individuals, even in areas with relatively high aggregate fish consumption, may still be undernourished. Inequalities within fishing communities [156] or even within the household [27,66] create varying levels of vulnerability within and between households. Efforts to reduce such inequalities and the negative resulting health outcomes among specific demographics, such as children and lactating women, include increased small fish consumption in Bangladesh [157], Cambodia [158,159], Malawi and Zambia [160,161].

Understanding these cultural and social elements are critical for contextualizing the science into meaningful food security policy and development recommendations. Belton et al. [126] provide a useful framework to conceptualise the thematic intersection of these cultural and social variables. These data need to now be contextualised in the broader diets of these populations and social categories must be disaggregated, as different solutions to micronutrient deficiencies will vary by demographic. Currently, limited literature exists around the potential for the use of aquatic animals to address local population micronutrient deficiencies [162]. Exemplary literature centred around particular micronutrient deficiencies in Cambodia and Bangladesh [163,164,165,166] could be combined with food pairing and dietary modelling as by Weidema and Stylianou [18] and applied to other contexts. Incorporation of fish into convenience products and fortified foods may be a useful strategy to overcome barriers to consumption among some groups [167]. Using locally available, calcium-rich small fish was found to be cheaper and more culturally acceptable in Cambodia than the normal dairy-based fortified supplements. More generally, even aquatic animal by-products rich in micronutrients [79,83] could be better utilised for direct human consumption through their inclusion in fortified processed foods than their use as ingredients in livestock feeds.

Sustainable food security solutions will require circular economy principles that minimise the waste of these micronutrients from the food system and consider the globalised nature of aquatic animal trade. This global trade in farmed aquatic animals is already highly dependent on flows of feed ingredients produced and traded internationally, a phenomenon that challenges the idea that ‘local’ is always the more sustainable choice.

### 2.6. Local Versus Global: Perceptions and Realities

The narrative that ‘locally-grown’ food has equivalence with sustainability has gained attention in North America and Europe: for some wealthy consumers, the fish products’ country of origin is an important factor when attempting to choose sustainable foods [139,168,169]. In reality, short food supply chains (SFSC) have both strengths and weaknesses in terms of their contribution to sustainability goals [170]. Two main reasons explain why this perception that ‘locally-sourced aquatic foods are more sustainable’ is problematic. Firstly, it is uninformed of the highly globalised nature of these foods’ production, where even aquatic products grown in North America and Europe rely on international trade for production inputs. For example, the majority of feed ingredients for ‘locally-grown’ Scottish salmon are from outside of the UK, and the product is traded to over 50 countries [52].

The second issue is that food miles, measured by GHG emissions from fuel consumption during transportation, are a poor proxy for sustainability [171]. Evidence on the GHG emissions produced across the supply chain suggests that for most food products, transportation accounts for less than 10%, whereas land use and farm-stage emissions account for more than 80% of total GHG emissions [172]. The carbon footprints of meals from imported aquatic animals were found to be similar to that of domestically produced aquatic animals, and sometimes lower, depending on the species consumed [173]. For the UK consumer, the environmental impacts of a fillet of farmed tilapia grown in Thailand might be lower than those of locally produced Scottish trout simply due to differences in edible yields and respective environmental impacts of feed used in the production of these two species [174]. While offshore salmon production has minimal direct land footprint, it has a very large footprint associated with the feed and chemicals used, and effluent from cages have their own associated environmental impacts [52]. The degree to which production is integrated within broader food landscapes, or is located apart as isolated systems, is critical to their characterisation.

Local, regional, or international trade in products significantly affects economic, environmental, and even nutritional outcomes [112]. While data on trade between and within the Global South are lacking, it appears that the small-to-medium-scale farms that dominate production in those areas are intensifying to supply local markets [5]. Such a trend may have implications for nutritional security and is also demonstrative of the aquaculture trade being increasingly ‘multi-polar’ between and within the Global South rather than a unilateral South-North flow of product as often characterised in the literature [175].

Export-oriented aquaculture in the Global South has been criticised for exporting nutrients from food-scarce environments [4]. In some contexts, an export orientation can lead to local trade-offs in nutritional security: for example, intensive monoculture of salmon in Chile has been criticised as an ‘extractive enclave economy’ [176], where undernourished people in local communities do not directly benefit from the salmon production due to failures in the broader food system and cultural and economic barriers to consumption. In contrast, some food systems, particularly in tropical contexts where there are undernourished people, remain highly diverse in terms of intensity and functionality of production, and diversity of species produced for both international and local markets. Shrimp production in Thailand is a classic example, where progression and diversification into products for domestic markets occurred after initial export-orientated development [177]. This has also occurred in Vietnam and Bangladesh [44,178,179] through evolution towards lower-input systems that are less risky and more opportunistic. Mamun et al. [66] identified that extensive polyculture of shrimp in Bangladesh, destined for processing and export, also resulted in a range of other aquatic animals that were consumed locally and greatly supported nutritional security. Even when considering export-oriented species such as shrimp, prawns, and pangasius, generally less than half of harvested biomass (the tails and fillet respectively) is exported: by-products including the nutrient-rich crustacean heads and claws are retained for local consumption [66], whereas pangasius by-products, which constitute a dominant proportion of harvested yield for this species, are recycled into nutritious pig feed in Vietnam.

As an example of these complexities, consider Nile tilapia. This fish is the fifth most popular aquatic animal food in the USA, and has long been promoted as a sustainable choice; it is, after all, a microphagous species, eating low in the food chain and perceived favourably to farmed carnivorous Atlantic salmon (second favourite) [180]. Same-sized fillets of the latter, however, had lower GHG emissions and scored better in other impact categories than the former [112]. This is partly explained by the higher fillet yield of salmon (over 60%) compared to tilapia (<40%) [55]. These results are compounded when compared nutritionally. Farmed salmon again scores better across a range of nutritional indicators compared to tilapia [13]. However, these rankings reflect tilapia being processed and prepared in the same way as salmon, which is not representative of practices in most consumer communities around the world, where the fish is eaten across a wide range of sizes and often with little processing or ‘plate waste’. For example, Chinese tilapia grown to a large individual size, filleted, and maybe dressed in breadcrumbs or a sauce and imported into the USA will have been fed a formulated plant-based diet, a proportion of which may well have been sourced from the USA, and with a significant carbon footprint and a nutrient profile reflecting its diet. In contrast, many consumers (i.e., especially in LMICs dependent on fish for food security) are eating small tilapias harvested from nutrient-rich ponds or public waters that have fed on naturally occurring plankton, and are eaten whole, which deliver much more favourable nutritional and sustainability outcomes. Backyard tilapia culture in such countries, such as Zambia, meets critical subsistence needs for small-scale producers and the broader community [181]. In contrast, intensively raised tilapia and, indeed, salmon exported as fillets for consumption in higher value markets typically contribute to diets that are already well-balanced and nutrient-dense, making their role in food and nutrition security less critical.

Systems thinking about the role of aquatic foods in sustainable food security is complex and requires interdisciplinary knowledge. Understandably, consumer knowledge and ability to make decisions around the environmental and nutritional impacts of farmed seafood and terrestrial alternatives is limited. Aquatic foods are further complicated by the fact they may be sourced from capture fisheries rather than farmed; we now consider this dichotomy, which is the basis of further misinterpretations and confusion.

### 2.7. Aquatic Animal Production Systems Span across the Fisheries—Aquaculture Continuum

European consumers’ understanding of the seafood production system typically values wild-caught aquatic animals higher than farmed based on taste or perceived safety [182,183,184]. This perspective is influenced by the overwhelmingly negative media framing of aquaculture, particularly in the Americas and Europe (e.g., misunderstandings around the use of antibiotics and contaminants [52,137,185,186]). At the point of sale, consumers typically remain ignorant or confused, often exacerbated by variable nomenclature, lack of labelling, or outright fraud [153,187,188,189]. Consumers are under constant pressure to make trade-offs between food products, for instance, on price and nutritional information [144]. Purchasing preferences may also be influenced by organoleptic properties (i.e., taste, smell, appearance), which vary between different species and production systems [190]. However, branding and knowledge are also important: while consumers show a preference for ‘wild’ fish, blind taste tests showed that the consumers preferred the organoleptic properties of farmed fish [183,190]. There is emerging evidence that farmed and wild fish are not equivalent in terms of their nutritional content and therefore their contribution to balanced diets is questioned; however, this discussion often becomes polarised as a misleading “wild is ‘good’ and farmed is ‘poor’” narrative.

On a global scale, published research on the nutritional outcomes of aquatic animal consumption is disproportionately focused on marine species e.g., [28,33,173,191], and the narratives around aquatic foods have become synonymous with marine environments and species [14,30,192]. SDG 14, ‘Life Below Water’, focuses exclusively on oceans and coastal systems, despite freshwater and inland areas dominating the rise in farmed production [31,32,34] and a sustained increased harvest from freshwater fisheries [193]. Inland fisheries, rarely monitored or included in national statistics, have been dubbed the ‘Hidden Harvest’; their important contribution to food and nutrition security is only now being realised [30,192]. This is in contrast to the known nutritional reliance of so many populations on freshwater inland fisheries for food security [32,193] and the fact that the majority of aquaculture operations are based in freshwater [9,31]. A recent analysis points to the imbalance in outputs from researchers based in the Global North, where marine systems and aquatic food sourcing predominate, compared to those in the Global South [194]. This dynamic may also be due to a lack of species-specific nutrient composition data, although this has recently begun to change with initiatives such as FishBase.se becoming available [77] and the Blue Food Assessment [13].

Meanwhile in policy discussions, aquaculture continues to be framed either positively, as a substitute for overexploited fisheries, or negatively, as a key pressure causing their failure through the trade in marine ingredients used in aquafeeds e.g., [195]; in reality, neither is an axiom precisely because of the diversity of these products and systems, as described throughout this paper. The use of small wild fish in aquafeeds is one clear example of the continuum. Critics point to continuing impacts of IUU (Illegal, Unreported and Unregulated) fisheries and trade in small wild fish caught in low-income countries with poor governance; these small wild fish are sold for aquafeeds, leading to detrimental impacts on local peoples’ nutritional security e.g., [28,34]. In response, aquaculture interests in Europe and North America point to the rapid improvement in efficiency of marine ingredient use such that fish in-fish out ratios (eFIFO) [196], the amount of wild fish used to produce an equivalent weight of farmed fish, has rapidly declined. Fewer fish are being used for marine ingredients as global prices have soared [196]. Global marine ingredients supplies are increasingly composed of aquatic animal by-products, which would not be directly consumed by people and have historically been wasted [79].

Framing ‘fisheries’, ‘aquaculture’, and indeed, agriculture as strictly different sectors is inappropriate given the high levels of both physical integration and nutrient flow between them. Many fish production systems are based in an aquaculture–fisheries continuum where there is often no clear binary [30]. One example is found in the floodplains of South and Southeast Asia, where farmers manage systems to ensure that both wild (‘self-recruiting species’) and hatchery species are produced together [95,197]. The increasing importance of various forms of culture-based fisheries [198,199,200] and sustained importance of capture-based aquaculture [201] suggest how interdependence remains critical to economic systems. Earthen pond systems in Zambia, for example, are made up of stocked fish but also fish that enter into ponds from the wild [181].

An emerging literature has linked large, farmed fish with lower nutritional content than small-sized, wild species; these findings were from Bangladesh, where farmed fish now contribute more than half of the total diet [95] and where one survey found more than 70% were now sourced from the market [150]. However, further scrutiny of the data suggests that there were wide differentials between small indigenous fish species in terms of key micronutrients and that comparisons of edible yield of individually small and large fish, irrespective of their origin, are problematic. Larger fish, regardless of their source, are often de-headed, gutted, and/or filleted in preparation for individual consumption, whereas small fish are often eaten whole, including the bones and viscera, which are rich in micronutrients. This occurs globally, from anchovies on a pizza in New York to fried mola fish in a curry sauce in Bangladesh.

Optimising nutritional sufficiency across the diet, and the role of aquatic foods, however, requires understanding of the micronutrient contributions from elsewhere in the diet. Market-based assessments ignore the dietary contribution from local sourcing which is still common [166], typically of smaller fish, both wild and farmed, that have lower sales value in markets. Furthermore, an escalating price gap [5] between wild (more expensive) and farmed (cheaper) affects the affordability of micronutrients derived from farmed and wild fish of various sizes.

## 3. The Way Forward

**Figure 2:**
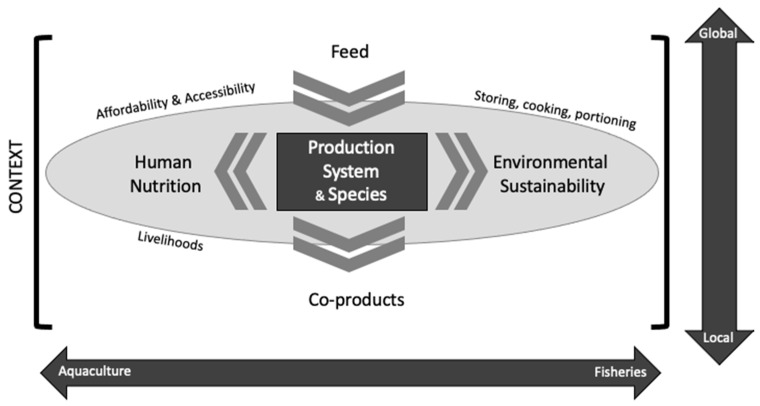
Framing of the role of aquatic animals in food systems, under current understandings of food systems theory, value chains, livelihoods, nutritional outcomes, and planetary boundaries thinking.

Aquatic animal systems discussions need to be informed by wider food systems literature to have better debates [202]. We have identified a specific need: because of the diversity, we need to include species- and production-level analysis that compares nutritional outcomes with environmental impacts contextualised by regional consumption preferences. Analysis could be modelled after and expanded on methods by, for example, Hallstrom et al. [29] and others, but informed by regional differences in edible portions, preparation practises, and distribution at the individual level. Furthermore, discussions should contextualise the role of nutrients from aquatic foods in the broader diet, as presented by Weidema and Stylianou [18], but also consider socio-cultural factors such as affordability, availability, and cultural preferences. Discussions must allow for the examination of different systems’ impact on different regions and populations: this will reflect (1) the highly international nature of the global seafood trade and the circular economy therein, and (2) all nutritional outputs of aquatic food systems, including co-products, rather than just the primary species. This framework is visualised in Figure 2.

Integrating more nuanced yet systematic discussions on aquatic foods into food security policy is important in streamlining consistent and evidence-based information to consumers. This will also help counteract the overwhelmingly negative and unbalanced public media portrayals of aquaculture or specific fish species. Ultimately, frameworks that truly consider the whole aquatic animal chain, within the broader food system will support food security goals as we strive to sustainably feed the planet.

## Data Availability

No new data were created or analyzed in this study. Data sharing is not applicable to this article.
